# Three-Dimensional Left Ventricle Strain Echocardiography in the Detection of Anthracycline Cardiotoxicity in Children with Acute Lymphoblastic Leukemia—Retrospective Case-Control Analysis and Relation to the Cardiotoxicity Risk Factors Assessment

**DOI:** 10.3390/cancers18172867

**Published:** 2026-09-04

**Authors:** Julia Haponiuk-Skwarlińska, Halszka Kamińska, Jakub S. Gąsior, Katarzyna Albrecht, Paweł Łaguna, Bożena Werner

**Affiliations:** 1 Department of Pediatric Cardiology and General Pediatrics, Doctoral School, Medical University of Warsaw, 02-091 Warsaw, Poland; 2 Department of Pediatric Cardiology and General Pediatrics, Medical University of Warsaw, 02-091 Warsaw, Poland; 3 Department of Pediatrics, Oncology, Hematology and Transplantology, Medical University of Warsaw, 02-091 Warsaw, Poland

**Keywords:** 3D strain echocardiography, speckle tracking, anthracycline cardiotoxicity, acute lymphoblastic leukemia, pediatric cardio-oncology

## Abstract

Background/Objectives: Acute lymphoblastic leukemia (ALL) treatment is effective but involves the use of cardiotoxic anthracyclines. Data on three-dimensional (3D) strain echocardiography utility in the monitoring of acute and early-onset cardiotoxicity in children with ALL are scarce. The aim was to assess the left ventricular function in 3D strain echocardiography in children with ALL treated with anthracyclines in reference to a healthy cohort and to assess risk factors of possible subclinical cardiotoxicity. Methods: For this study, medical records from between May 2023 and December 2024 were retrospectively analyzed, and the echocardiographic data were reassessed. The study group consisted of children diagnosed and treated for ALL for at least 6 months with the use of daunorubicin and doxorubicin (or only with daunorubicin) who underwent routine cardiological evaluation with 3D echocardiography in >6 months and <2 years from the beginning of treatment. The echocardiographic records were reassessed offline to analyze the left ventricle 3D global longitudinal (GLS 3D), circumferential (GCS 3D) and radial (GRS 3D) strain. The results were compared with the results of 24 apparently healthy peers matched by sex and age. A set of models was developed to test literature-based subclinical cardiotoxicity determinants in relation to the obtained 3D strain values. Results: The study group consisted of 42 children (22 males and 20 females) aged 2–17 years (mean 7.7 ± 4.1 years) treated for ALL. The median time from the first anthracycline dose was 0.59 (IQR1 = 0.5; IQR3 = 0.96) years with a cumulative equivalent anthracycline dose of 158.2 ± 78.0 (min. 48.0–max. 451.8) mg/m^2^. Statistically significant differences between study and control groups were observed in terms of LVEF 3D (53.9 ± 4.4% vs. 59.1 ± 5.8%, *p* < 0.001), GLS 3D (−21.8 ± 4.2% vs. −25.2 ± 3.6%, *p* < 0.001), GCS 3D (−22.3 ± 3.8% vs. −27.3 ± 4.7%, *p* < 0.001) and GRS 3D (37.1 ± 5.0% vs. 44.6 ± 5.7%, *p* < 0.001). None of the previously described cardiotoxicity risk factors—age at the diagnosis, sex, age at treatment initiation, body surface area (BSA) at treatment initiation, time since treatment initiation, ALL subtype, total anthracycline dose and doxorubicin treatment—were statistically significant in the GLS or GCS models. Conclusions: GLS 3D, GCS 3D and GRS 3D of the left ventricle may be sensitive indicators of myocardial dysfunction in children with ALL treated with anthracyclines. The relation of the previously described cardiotoxicity risk factors to the 3D strain values and myocardial dysfunction is inconclusive; further studies are warranted.

## Simple Summary

Anthracyclines are very effective in the treatment of acute lymphoblastic leukemia (ALL) but unfortunately cardiotoxic. Our study is the first to report comprehensive 3D echocardiography with strain assessment in the population of children treated for ALL to compare it with healthy individuals and to analyze previously described cardiotoxicity risk factors in relation to the 3D-echocardiography-detected cardiac function impairment in this group of patients. As ALL is the most common childhood malignancy with a promising prognosis, we strongly believe that the early detection of cardiotoxicity before the onset of heart failure symptoms is imperative for the implementation of cardioprotection in this population.

## 1. Introduction

Among all pediatric malignancies, acute lymphoblastic leukemia (ALL|) is the most common [1]. Five-year event-free survival (EFS) and estimated 10-year overall survival (OS) for childhood ALL currently approaches 90% in developed countries [2,3]. The main protocols of leukemia treatment include the use of steroids, vincristine, asparaginase and anthracyclines [4]. Although anthracycline therapy is highly effective, it is also the most recognized cardiotoxic cancer treatment among the various cancer treatments responsible for chemotherapy-related cardiac dysfunction (CTRCD) [5,6].

Cardiotoxicity can be caused by many mechanisms, including cardiomyopathy/heart failure, coronary artery disease, stroke, pericardial disease, arrhythmias and valvular and vascular dysfunction [5,7]. Anthracycline therapy is the most common cause of chemotherapy-induced cardiomyopathy (CCM), with a clinical presentation spectrum from acute to early-onset and late-onset [5]. Current research focuses on subclinical cardiotoxicity—early myocardial injury from cardiotoxic therapy, detectable by sensitive imaging/biomarkers leading to the functional consequence—a subclinical cardiac function impairment before the onset of heart failure symptoms with preserved left ventricle ejection fraction [6,8]. Many cardiotoxicity preventive methods are now studied in adults and during in vitro studies [9]. Therefore, the early and accurate detection of cardiotoxicity is essential to prevent, delay or treat cardiac injury or advanced heart failure.

Currently, strain/speckle-tracking echocardiography is recommended and used to determine regional and global myocardial dysfunction in adult patients in response to cardiotoxic chemotherapy [6]. Global longitudinal strain (GLS) in 2-dimentional speckle-tracking echocardiography (2D-STE) is the mostly reviewed parameter [10,11,12,13,14,15,16]. Most of the previous studies on cardiotoxicity detection after ALL treatment focus on late-onset myocardial changes in adult childhood cancer survivors [11,13,17].

Three-dimensional speckle-tracking echocardiography (3D-STE) is an advanced imaging technique of higher feasibility and reproducibility over the two-dimensional technique [18]. 3D-STE is not yet prevalent in the monitoring of acute and early-onset cardiotoxicity in children treated for ALL. The literature on 3D-STE is scarce; preliminary data on 3D-STE’ in 32 children with ALL suggest possible decline in GLS-3D and GCS-3D pre- vs. post-anthracyclines use in the induction and intensification phase of the treatment [19].

**Aims**

1. To assess the left ventricular function in 3D strain echocardiography in children with ALL treated with anthracyclines in comparison to healthy individuals.
2. In order to thoroughly assess 3D strain utility in the cardiotoxicity surveillance, we aimed to analyze previously described cardiotoxicity risk factors in relation to the 3D-echocardiography strain results.

## 2. Materials and Methods

### 2.1. Participants

**Figure 1:**
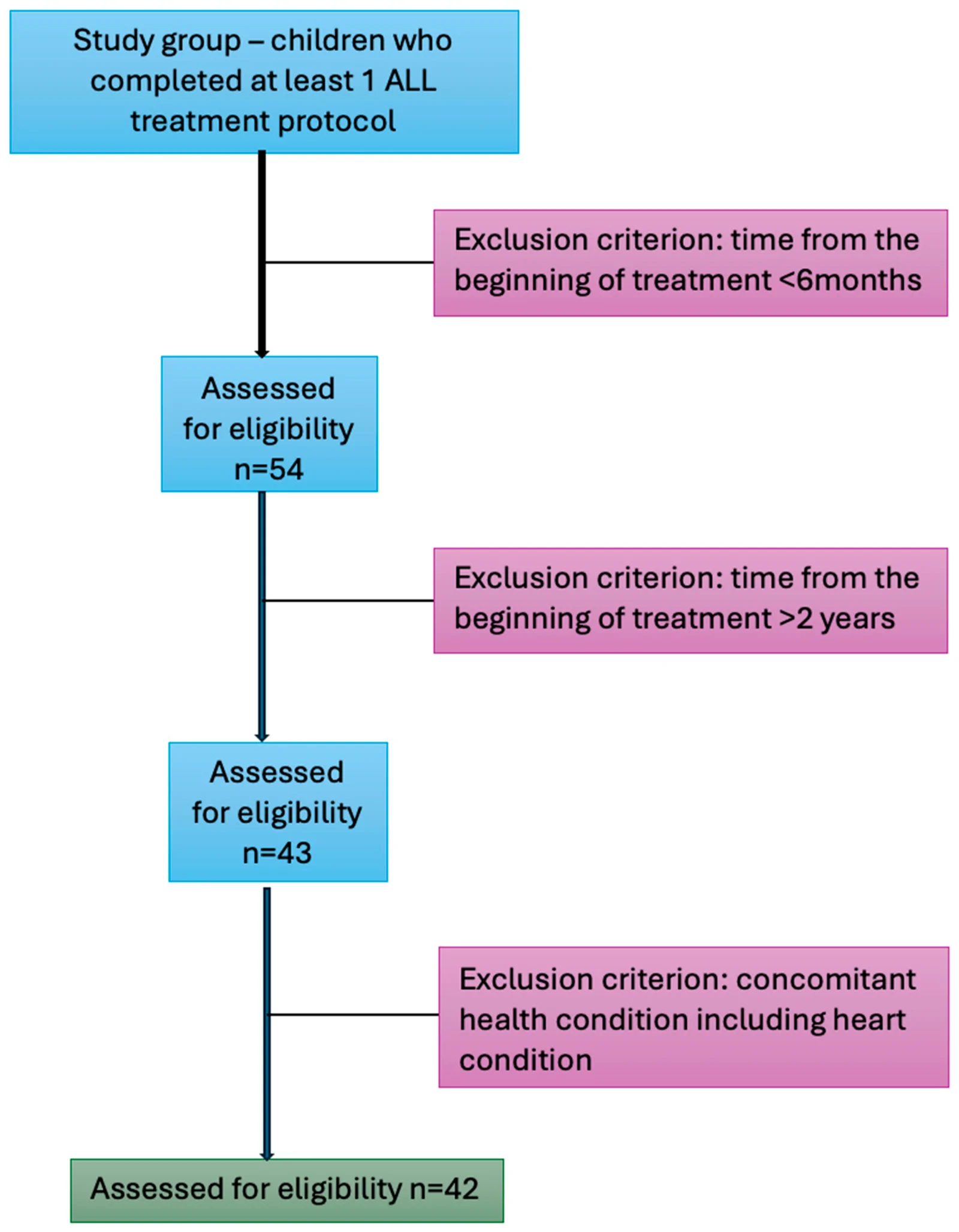
Study group inclusion and exclusion criteria.

For this study, medical records and echocardiographic studies from between May 2023 and December 2024 were retrospectively analyzed and reassessed. The children diagnosed with ALL and treated at one oncology department who were routinely evaluated at the echocardiography laboratory of the pediatric cardiology department were chosen for the study group. In total, 54 consecutive children who completed at least 6 months of anthracycline therapy for ALL were considered and recruited to the study group according to the inclusion and exclusion criteria (Figure 1). For this study, time from the beginning of the anthracycline therapy was defined as the follow-up time. Patients who were treated with other potentially cardiotoxic methods (radiotherapy or hematopoietic cell transplantation) were excluded. In order to analyze only children after anthracyclines use but during the ALL therapy, participants whose follow-up was shorter than 6 months and longer than 2 years were excluded from the study group. One child was excluded from the study group after their medical records analysis due to a previous diagnosis of congenital heart disease.

To provide a reference for the echocardiographic assessment, a control group of 24 healthy children matched by sex and age was included. The controls were children referred for the echocardiography in which cardiovascular disease, significant systemic disease, congenital heart disease or other conditions expected to affect cardiac structure or function were excluded and discharged in good health.

The study was retrospective and based on available clinical and echocardiographic records in the sampling period; therefore, a conventional prospective sample-size calculation was not performed before data collection.

### 2.2. Practical Conduction of the Study

Medical Record Abstraction

Demographic information, including age at diagnosis, sex, body mass and weight, age at treatment initiation and length of follow-up, was collected, along with patient data on treatment details. There were no more than 8.6% of missing retrospective medical data in each variable. Anthracyclines included doxorubicin and daunorubicin.

The 0.6 cardiovascular toxicity dose ratio was used for the daunorubicin equivalence and cumulative anthracycline dose calculation [6,20].

Echocardiography records

All echocardiographic exams were performed according to the international recommendations on an EPIQ CVx 5.0 ultrasound machine (Philips Medical System, Cambridge, MA, USA) using an X5 transducer from at least 2 cardiac cycles. Sedation was not used.

The echocardiographic records were reassessed after the echocardiographic exam. Before the reassessment, the echocardiographic records were blinded from the patient data, except for age and sex. For the 3D records in our protocol, the acquisition parameters were standardized according to the patient’s heart rate. The frame/volume acquisition rate was selected to remain comparable to the patient’s heart rate, with a difference of no more than approximately 20 beats/min, in order to obtain adequate temporal resolution.

Full-volume datasets were acquired using two cardiac cycles in cooperative/stable examinations and one cardiac cycle when the child was moving, which was necessary to minimize motion-related artifacts.

**Figure 2:**
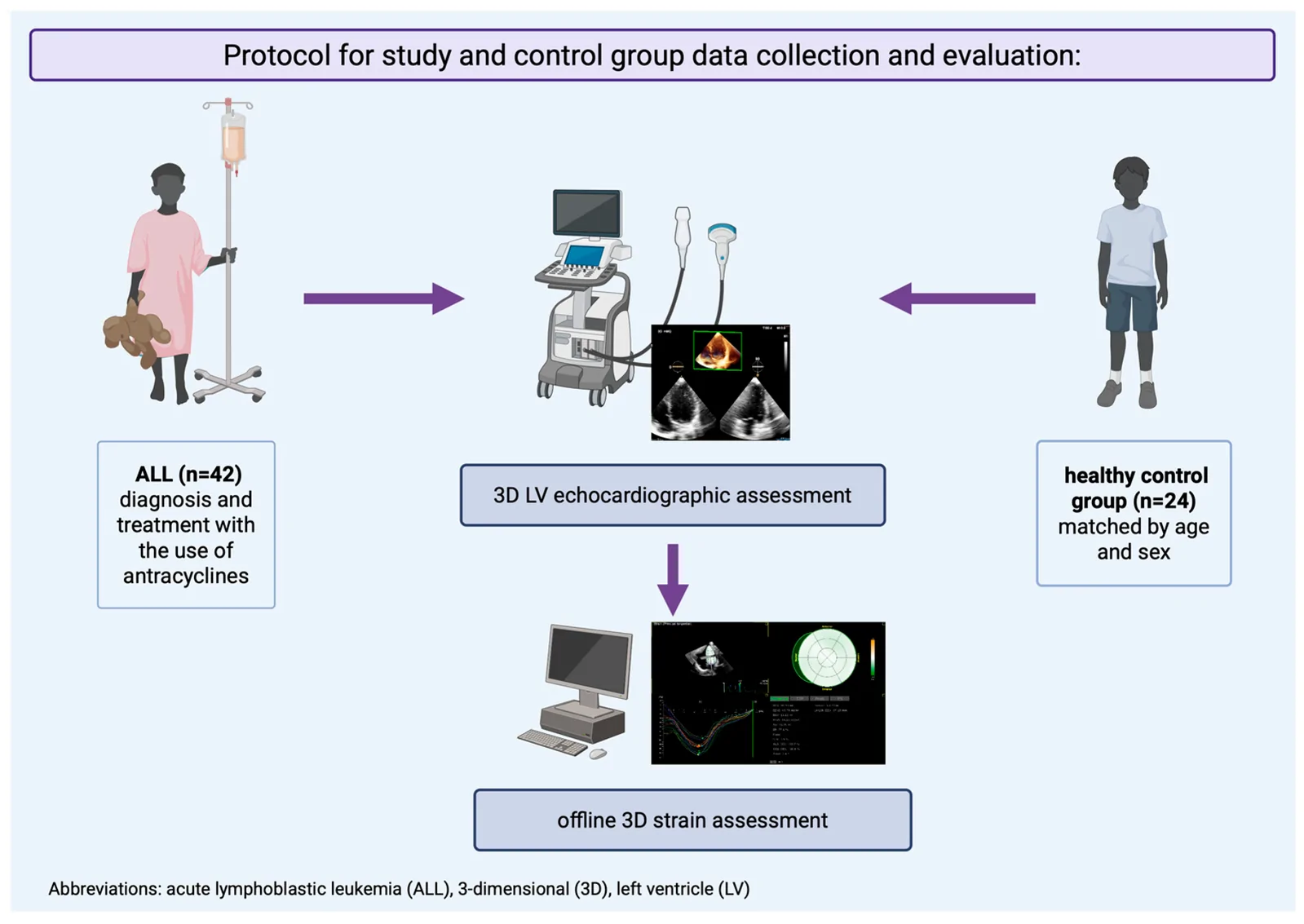
Figure to present the protocol for study and control group echocardiographic data collection. Created in BioRender. Haponiuk, J. (2026).

3D echocardiographic records were analyzed offline with the use of 4D LV-Analysis software UWS (Ultrasound Work Space/TomTec) 6.0 version (Philips Medical System, Cambridge, MA, USA), assessing left ventricle ejection fraction (LVEF 3D), end diastolic volume (EDV 3D), end diastolic volume indexed (EDVI 3D), end diastolic length (ED length), end systolic volume (ESV 3D), end systolic volume indexed (ESVI 3D), global longitudinal strain (GLS 3D), global circumferential strain (GCS 3D) and radial strain (GRS 3D). Also, parameters measured by the software were collected—torsion and twist of the left ventricle. No manual contour correction was performed. The protocol for echocardiographic data collection is presented in Figure 2. All examined children who met the inclusion and exclusion criteria for the study group and for the control group had 3D echocardiographic records of adequate quality to be reassessed with the offline software. The controls underwent echocardiographic assessment according to the same standardized protocol. All recordings were reviewed by two experienced echocardiographers providing intra and interobserver reliability.

Statistical Analysis

Statistical analysis was performed using IBM^®^ SPSS^®^ version 30.0.0. Medians, proportions and percentages were used to describe the population and resulting observations. The comparisons between groups were performed with the T-test for normally distributed data and the U-Mann Whitney test for non-normally distributed data. The interobserver and intraobserver variabilities were checked with the T-test as well as the Spearman correlation with a 95% confidence interval.

- Regression analysis

Multivariable ordinary least-squares linear regression models were fitted for global longitudinal strain (GLS [3D]), global circumferential strain (GCS [3D]) and global radial strain (GRS [3D]). The determinants entered simultaneously were age at the diagnosis, sex, age at treatment initiation, body surface area (BSA) at treatment initiation, time since treatment initiation, ALL subtype, total anthracycline dose and doxorubicin treatment. Two missing total anthracycline doses were reconstructed from the recorded protocol-I daunorubicin dose; therefore, all models included 41 participants. The results are presented as unstandardized regression coefficients (B), 95% confidence intervals (CI), standardized coefficients (β) and two-sided *p*-values. Model fit was assessed with R^2^, adjusted R^2^, the F test and residual root mean square error (RMSE). A two-sided *p*-value below 0.05 was considered statistically significant.

The significance level was set at α = 0.05 for all applied tests.

Ethical committee approval

This study was conducted according to the guidelines laid down in the Declaration of Helsinki, and all procedures involving patients’ data were approved by the local Ethic Examining Committee of Human Research of the Medical University (Decision of Approval: AKBE/63/2025 from 24 February 2025).

## 3. Results

A total of 42 children were included in the study group (Figure 1), and 24 healthy children were enrolled in the control group. The mean age at the assessment was 7.7 ± 4.1 years (min. 2 years, max. 17 years); there were 22 males and 20 females. Most of the patients presented with ALL type C (common, B type) (*n* = 34, 80%) and were treated with the AIEOP BFM 2017 protocol [21] (*n* = 35, %). All of the participants were after induction and intensification therapy and during the maintenance stage of treatment and were initially classified as a low-risk patients (M1 response) in 88% (*n* = 37). The median time from the first anthracycline dose was 0.59 (0.50; 0.96) years (min. 0.5 year, max. 2.0 years). A total of 42 (100%) patients were treated with doxorubicin and daunorubicin, and 4 patients were treated only with daunorubicin. The cumulative equivalent anthracycline dose [6] was 158.2 ± 78.0 (min. 48.2–max. 451.8) mg/m^2^, and 8 (20%) children obtained a cumulative equivalent anthracycline dose of >200 mg/m^2^ (Table 1).

The baseline characteristics of the control group and matching are presented in Table 2.

There was a statistically significant difference in LVEF 3D as well as in GLS 3D, GCS 3D and GRS 3D between the study and control groups (Table 3). All strain mean values were observed as ≥−21.0% in children treated for ALL. The volumes and lengths of the left ventricle were not statistically significantly different, except for ESVI 3D. There was no difference observed in the twist and torsion of the left ventricle. Next, the LVEF 3D was <50% in 5 (11%), <55% in 28 (60%) and >55% in 14 (29%) members of the study group (Table 4). The echocardiographic records were checked for intra- and inter-observer variability by two experienced echocardiographers. The statistical analysis showed a highly compatible result (*p*-value above 0.915 for all of the checked data). The interobserver and intraobserver reliability for LVEF 3D and strain values are presented in Table 5.

Regression models analysis of the cardiotoxicity risk factors

The GLS model explained 21.7% of GLS variability (R^2^ = 0.217; adjusted R^2^ = 0.021), but the complete model was not statistically significant (F = 1.11, *p* = 0.384). The GCS model explained 22.2% of GCS variability (R^2^ = 0.222; adjusted R^2^ = 0.027) and was not statistically significant (F = 1.14, *p* = 0.365). The GRS model explained 28.9% of GRS variability (R^2^ = 0.289; adjusted R^2^ = 0.112) and was likewise not statistically significant (F = 1.63, *p* = 0.156) (Table 6).

Individual statistically significant associations are displayed in Table 7. Within the GRS model, a higher current age was associated with lower GRS (B = −0.85 per year, 95% CI −1.64 to −0.06, β = −0.71, *p* = 0.035). Total anthracycline dose was positively associated with GRS (B = 0.03 per mg/m^2^, 95% CI 0.01 to 0.06, β = 0.64, *p* = 0.013). No determinant was statistically significant in the GLS or GCS models at the 0.05 level.

## 4. Discussion

Our study is the first to report comprehensive 3D echocardiography with strain assessment in the population of children with ALL to compare it with healthy individuals and to test literature=based subclinical cardiotoxicity determinants in relation to the obtained 3D strain values.

### 4.1. Results vs. Normal Values

This retrospective study showed that left ventricle strain parameters, including GLS 3D, GCS 3D and GRS 3D in children treated for ALL (0.5–2.0 years from treatment initiation, within the maintenance phase of the treatment), although within reported normal values [23], have significantly lower absolute values than in a healthy cohort. In our study, all 3D mean strain values were equal or above −21.0% in both groups [23]. GCS 3D and GRS 3D normal values of LV strain as well as twist and torsion parameters using 3D STE have not been established in a large group of normal subjects. The normal values proposed by Kaku et al. for GCS 3D are −28.9 ± 4.6%, and for GRS 3D, they are 88.0 ± 21.8% [24], which matches other literature values [23,25] as well as the values observed in our control group. Although the observed mean GLS 3D value in the study group was within the literature norms, the GCS 3D as well as the GRS 3D had lower absolute values.

Next, our study group also presented with lowered LVEF 3D in comparison to controls, with a mean of 53.9 ± 4.4% and <50% in 5 (12%) and of <55% in 25 (60%) participants. The presence of a drop in EF is a cardiotoxicity criterion [6]; however, 90% of the study group presented with an LVEF 3D above 50% with abnormal strain parameters detecting subclinical cardiotoxicity. Also, LVEF 2D reference intervals should not automatically be applied to LVEF 3D [26] as it is device/software-dependent. In our study, LVEF 3D was significantly lowered in children following anthracycline therapy from our reference control group assessed under the same conditions.

### 4.2. Cardiotoxicity Detection

So far, literature data are not consistent regarding the echocardiographic indicators of early-onset cardiotoxicity among children treated for malignances. According to a literature review by Kouwenberg et al., systolic impairment is defined as a decrease in fractional shortening and/or ejection fraction as well as a reduction in global longitudinal strain (absolute value) in two-dimensional echocardiography (GLS 2D), while diastolic dysfunction is defined as an increase in the Tei index or a decrease in the E/A ratio [27]. 2D speckle tracing echocardiography including GLS 2D in children undergoing ALL treatment was first described in the literature as a more sensitive method than conventional 2D echocardiography in the detection of early cardiotoxicity [12,16,27]. Militaru et al. evaluated children before and 3 months after the initiation of ALL therapy and observed a significant drop in LVEF 2D and GLS 2D (absolute value) [14]. GLS 2D is also reported as an impaired parameter in childhood cancer survivors [11,13,15,28]. Other studies report right ventricle impairment in long-term survivors of childhood ALL [29]. 2D and 3D strain echocardiography had been evaluated in adults receiving chemotherapy as well as in childhood cancer survivors and is recognized as a sensitive method of cardiac function assessment [30,31]. Diastolic impairment measured with left atrial strain has also been reported in adult childhood ALL survivors [28]. Since 2022, the European Cardiac Society recommends LVEF assessment, preferably with the use of 3D echocardiography, as well as GLS 2D assessment in adult patients before and after anthracycline therapy (especially within the first 12 months after treatment completion) for the detection of early function impairment of the left ventricle [6,32]. In 2022, the Australian and New Zeeland Delphi Consensus developed the first individualized cardio-oncology recommendations for pediatric oncology patients basing on adult guidelines [29]. According to the guidelines, 3D echocardiographic measurements of LVEF with GLS are the optimal recommendation for surveillance. We had already preliminarily assessed the 3D strain in a prospectively collected group of children with ALL (*n* = 32) before the initiation of the treatment and after the induction and intensification phase of the treatment (after around 7 months)—the GLS 3D and GCS 3D showed statistically significant difference between pre- and post-treatment, and the GRS changes were not significant [19]. All examined children who met the inclusion and exclusion criteria for the study group and for the control group had 3D echocardiographic records of adequate quality to be reassessed with the offline software. Thus, the 3D imaging in our cohorts seemed to be a feasible and achievable element of echocardiographic evaluation and is a promising complementary technique for detecting early myocardial deformation abnormalities.

However, no data on comprehensive three-dimensional (3D) strains’ utility in the monitoring of the children treated for ALL had been published, and our report is the first, as of the date of manuscript submission, to describe it. Further validation, preferably through prospective and longitudinal studies, is required before it can be considered a routine screening standard.

### 4.3. Cardiotoxicity Risk Factors in Relation to the 3D Strain

We have addressed several possible determinants of 3D-echocardiography-detected cardiac function impairment within the study group. Our population was assessed 0.59–2.0 years after anthracycline treatment initiation. The assessed strain value determinants included sex, age at diagnosis, age at the assessment, cumulative equivalent anthracycline dose [6], anthracycline used (doxorubicin, daunorubicin) time from treatment initiation, ALL type and BSA at the diagnosis.

In the population studied by us, only eight children exceeded a dose of 200 mg/m^2^, and two exceeded 300 mg/m^2^; these high doses were not related to observed strain values. Anthracyclines chemotherapy is considered a high cardiovascular risk factor among adult oncological patients for CTRCD. The cumulative equivalent dose of >300 mg/m^2^ is considered a potentially high risk dose for early-onset cardiotoxicity in adults and a cumulative anthracycline dose in general is considered an important risk factor of cardiotoxicity in children [27]. The Australian and New Zealand Delphi Consensus defines a patient as a high-risk for cardiac complications with a total cumulative dose of ≥250 mg/m^2^ [33].

In our analysis, after accounting for the number of determinants included, the models demonstrated limited explanatory power.

Within the GRS model, a higher age at the assessment was associated with a lower GRS value. The total anthracycline dose was positively associated with the GRS value. No determinant was statistically significant in the GLS or GCS models at the 0.05 level. Individual coefficient estimates should be interpreted cautiously because the complete GRS model was not significant.

We conclude that, based on our data risk factors for 3D strain values, a decrease cannot be clearly defined in our population, and further studies are necessary to confirm our findings. According to the literature risk factors for acute (within a week) and early-onset (within a year) cardiotoxicity in children, most reported in the literature are older (>2 years old or >4 years old) [34,35], have Afro-American ethnicity and have cumulative anthracycline [27]. Different cumulative anthracycline doses are reported as the cut-off for an early cardiotoxicity risk—from >120 to >250 or above 300 mg/m^2^ [6,27,33]. Other early-onset cardiotoxicity factors that were assessed—sex, ethnicity, weight category, cytogenetic risk group, risk stratification according to the treatment protocol, initial white blood count, randomized treatment arm, microbiologically documented bloodstream infection during treatment, age ≥ 11 years category and other race—were not statistically related to the cardiotoxicity risk [34,35].

As ALL is the most common childhood malignancy with promising prognosis, we strongly believe that the early detection of cardiotoxicity before the onset of heart failure symptoms is imperative for the implementation of cardioprotection in this population [33].

### 4.4. Limitations

This study is of a retrospective nature based on the average population of Caucasian children affected by ALL. Although we focus on anthracyclines as the main factor of cardiac impairment, leukemia treatment, in multiple potential ways, can affect the cardiopulmonary fitness of these pediatric patients, including anemia, electrolyte imbalance, marasmus, lowered physical activity, treatment complications, etc., which is very prevalent in this group of patients and impossible to exclude as an additional risk. Deformation is a load-dependent variable. This is a pediatric population, and any medical examination in this particular group may lead to tachycardia or blood pressure alterations. It is universal for both the control and the study group, although we could see the statistically significant difference in strain parameters. Reproducibility relied primarily on correlation-based analyses for reproducibility assessment, not on the statistical tests. Because of the specific, narrow group of patients, our study does not consider other cardiotoxic treatment methods including irradiation of the chest region or VEGF, mTOR and BRC-Abl kinase inhibitors use. Further follow-up as well as a comparison of the echocardiography before and after anthracycline therapy could also provide an insight into the cardiac function through the process of treatment and convalescence.

## 5. Conclusions

- GLS 3D, GCS 3D and GRS 3D of the left ventricle may be sensitive indicators of myocardial dysfunction in children with ALL.
- 3D strain echocardiography may represent a useful complementary tool for detecting early myocardial dysfunction in pediatric patients exposed to anthracyclines and warrants further prospective evaluation.
- The data on 3D strain values determinants in the population of children treated for ALL are inconclusive and further research is warranted

## Figures and Tables

**Table 1:** Table with baseline characteristics of the study group.

Study Group Characteristics (n = 42)	
**age at diagnosis (years old)**	6.4 ± 3.5 (min. 1.7 years old, max. 17.0 years old)
**age at the assessment (years old)**	7.7 ± 4.1 (min. 2 years old, max 17 years old)
**sex**	22 (52%) males, 20 (48%) females
**ethnicity**	Caucasian n = 42 (100%)
**diagnosis**	34 (80%) ALL type C (common, B type), 8 (20%) ALL type T
**white blood cells at diagnosis**	17.15 × 10^3^ (4.7 × 10^3^; 33.3 × 10^3^) Normal WBC value n = 5 (12%) Leukopenia n = 12 (29%) Leukocytosis n = 25 (59%)
**percentage of blasts detected in manual count (%)**	90.6 (77.6; 94.5)
**treatment protocol**	AIEOP BMF 2017 [21] n = 35 (83%) ALL IC-BMF 2009 [22] n = 4 (10%) Other n = 3 (7%)
**stage of the treatment**	Maintenance n = 42 (100%)
**initial treatment response**	M1 n = 37 (88%) M2 n = 3 (8.5%) M3 n = 2 (6.5%)
**BSA at diagnosis**	0.89 ± 0.28
**BMI at diagnosis**	16.2 ± 2.0
**BMI percentile at diagnosis**	48 (21.0; 65.0)
**type of anthracycline used**	Doxorubicin n = 38 (90%) Daunorubicin n = 42 (100%)
**time from the first anthracycline dose (years) i.e., follow-up time**	0.59 (0.50; 0.96) min 0.5–max 2.0
**cumulative equivalent anthracycline dose (mg/m^2^)**	158.2 ± 78.0 min. 48.0–max. 451.8
**cumulative equivalent anthracycline dose >200 mg/m^2^**	n = 8 (20%)
**cumulative equivalent anthracycline dose >300 mg/m^2^**	n = 2 (5%)
**cumulative doxorubicin dose (n = 38) (mg/m^2^)**	98.4 ± 70.3 min. 30.8–max. 387.0
**cumulative daunorubicin dose (n = 42) (mg/m^2^)**	109.5 ± 34.4 min. 61.2–max. 226.8

Abbreviations: minimum (min), maximum (max), acute lymphoblastic leukemia (ALL), white blood cells (WBC), International collaborative treatment protocol for children and adolescents with acute lymphoblastic leukemia (AIEOP BMF 2017) [21], Randomized Trial of the I-BFM-SG for the Management of Childhood non-B Acute Lymphoblastic Leukemia (ALL IC-BMF 2009) [22], marrow, <5% blasts (M1); marrow ≥5 to <25% blasts (M2), marrow, ≥25% blasts (M3), body surface area (BSA), body mass index (BMI).

**Table 2:** Table with baseline characteristics of the matched reference group.

Parameter	Study Group	Control Group	*p* Value
age	7.7 ± 4.1 (min. 2 years old, max 17 years old)	7.2 ± 3.1 (min. 1.5–max. 11.9 years old)	0.619
Sex	22 (52%) males, 20 (48%) females	13 males (54%), 11 females (46%)	0.580

**Table 3:** Table showing the 3D LV systolic function in the study and control groups.

Variable	Study Group—Children with ALL n = 42	Control Group—Healthy Children n = 24	*p* Value	Mean Between-Group Difference (Absolute Value)	95% Confidence Interval (Lower/Upper)
EDV 3D (mL)	57.87 (43.2; 87.6)	65.7 (39.2; 90.4)	0.661 ^w^	0.7	−16.6/18.0
EDVI 3D (mL/m^2^)	61.5 ± 16.7	63.5 ± 12.2	0.142 ^t^	1.9	−9.8/5.9
ESV 3D (mL)	27.8 (18.7; 39.9)	26.1 (17.3; 34.0)	0.430 ^w^	5.9	−3.2/15.1
ESVI 3D (mL/m^2^)	29.7 (24.4; 34.3)	25.4 (20.2; 30.2)	0.022 ^w^*	3.9	−0.4/8.2
LVEF 3D (%)	53.9 ± 4.4	59.1 ± 5.8	<0.001 ^t^*	5.8	−8.1/−3.5
Length (ED) 3D (mm)	66.0 (62.0; 79.6)	67.6 (59.1; 81.0)	0.946 ^w^	0.2	−5.3/5.8
GLS 3D (%)	−21.88 ± 4.2	−25.2 ± 3.6	<0.001 ^t^*	2.8	1.0/4.6
GCS 3D (%)	−22.3 ± 3.8	−27.3 ± 4.7	<0.001 ^t^*	4.9	−2.9/6.8
GRS 3D (%)	37.1 ± 5.0	44.6 ± 5.7	<0.001 ^t^*	6.8	−9.19/−4.5
Twist (**°**į)	8.1 (3.9; 10.4)	8.8 (5.9; 12.4)	0.306 ^w^	1.281	−3.7/1.1
Torsion (**°**į/cm)	1.0 (0.5; 1.54)	1.24 (0.8; 1.63)	0.264 ^w^	0.043	−0.4/0.3

Basic statistics, for quantitative variables—mean ± standard deviation or median (first quartile; third quartile), w—Wilcoxon rank sum test, t—Student’s *t*-Test. * is for statistically significant values. Abbreviations: Acute lymphoblastic leukemia (ALL), 3-dimensional (3D), end diastolic volume (EDV), end diastolic volume index (EDVI), end systolic volume (ESV), end systolic volume index (ESVI), left ventricular ejection fraction (LVEF), end diastolic (ED), global longitudinal strain (GLS), global circumferential strain (GCS), global radial strain (GRS).

**Table 4:** Table showing number and percentage of participants according to the LVEF 3D result.

*LVEF 3D—Study Group*	*N (%)*
<50%	5 (12%)
<55%	25 (60%
>55%	12 (28%)

Abbreviations: 3-dimensional (3D), left ventricular ejection fraction (LVEF).

**Table 5:** Table showing interobserver and intraobserver variability for LVEF 3D, GLS 3D, GCS 3D and GRS 3D *p* value from t-test and correlation coefficient (R) with 95% confidence interval.

	*Interobserver*		*Intraobserver*	
*parameter*	*p* value	R, 95% confidence interval	*p* value	R, 95% confidence interval
*LVEF 3D (%)*	0.915	0.932, 0.732–0.984	0.936	0.986, 0.920–0.997
*GLS 3D (%)*	0.924	0.934, 0.738–0.985	0.968	0.986, 0.923–0.998
*GCS 3D (%)*	0.920	0.982, 0.925–0.996	0.952	0.981, 0.895–0.997
*GRS 3D (%)*	0.929	0.978, 0.906–0.995	0.926	0.976, 0.864–0.996

Abbreviations: 3-dimensional (3D), left ventricular ejection fraction (LVEF), global longitudinal strain (GLS), global circumferential strain (GCS), global radial strain (GRS).

**Table 6:** Overall fit of the multivariable linear regression models.

Outcome	N	R^2^	Adjusted R^2^	F Statistic	Model *p*-Value	RMSE	Residual df
GLS (3D)	41	0.217	0.021	1.11	0.384	4.22	32
GCS (3D)	41	0.222	0.027	1.14	0.365	3.79	32
GRS (3D)	41	0.289	0.112	1.63	0.156	4.72	32

R^2^ indicates the proportion of variability in the dependent variable explained by the full model. RMSE, root mean square error.

**Table 7:** Determinants (previously described cardiotoxicity risk factors) of 3D strain parameters in multivariable linear regression models.

Determinant	GLS (3D)	GCS (3D)	GRS (3D)
Age at the assessment [years]	B = 0.57 (−0.06 to 1.20); β = 0.55; *p* = 0.076	B = 0.38 (−0.19 to 0.95); β = 0.41; *p* = 0.179	B = −0.97 (−1.68 to −0.26); β = −0.80; *p* = 0.009
Sex (1-male vs. 0-female)	B = 1.03 (−1.83 to 3.89); β = 0.12; *p* = 0.468	B = 0.69 (−1.88 to 3.26); β = 0.09; *p* = 0.587	B = −0.67 (−3.86 to 2.53); β = −0.07; *p* = 0.675
Age at the diagnosis [years]	B = −0.17 (−1.33 to 0.99); β = −0.14; *p* = 0.771	B = −0.37 (−1.41 to 0.68); β = −0.33; *p* = 0.480	B = 0.42 (−0.88 to 1.72); β = 0.29; *p* = 0.517
BSA at the diagnosis [m^2^]	B = 0.75 (−12.99 to 14.48); β = 0.05; *p* = 0.912	B = −1.34 (−13.69 to 11.01); β = −0.10; *p* = 0.827	B = 1.46 (−13.92 to 16.83); β = 0.08; *p* = 0.848
time from the first anthracycline dose (years) i.e., follow-up time	B = −1.14 (−3.91 to 1.63); β = −0.16; *p* = 0.407	B = −1.43 (−3.92 to 1.06); β = −0.22; *p* = 0.252	B = 2.27 (−0.83 to 5.37); β = 0.27; *p* = 0.146
ALL type (1–B type vs. 0–T type)	B = 1.53 (−2.57 to 5.63); β = 0.14; *p* = 0.453	B = −3.23 (−6.92 to 0.46); β = −0.34; *p* = 0.084	B = 1.06 (−3.53 to 5.65); β = 0.08; *p* = 0.643
cumulative equivalent anthracycline dose [mg/m^2^]	B = −0.02 (−0.04 to 0.00); β = −0.42; *p* = 0.090	B = −0.02 (−0.03 to 0.00); β = −0.40; *p* = 0.101	B = 0.03 (0.01 to 0.05); β = 0.61; *p* = 0.012
doxorubicin in therapy (1- yes vs. 0–n0)	B = 0.79 (−4.02 to 5.59); β = 0.06; *p* = 0.740	B = 1.29 (−3.03 to 5.61); β = 0.11; *p* = 0.547	B = −3.10 (−8.47 to 2.28); β = −0.20; *p* = 0.250

Values are B (95% CI), standardized β and *p*-value. For binary variables, the coefficient compares category 1 against category 0, as coded in the original dataset.

## Data Availability

The data obtained in the study can be shared upon request.

## Abbreviations

The following abbreviations are used in this manuscript:

ALL	acute lymphoblastic leukemia
3D	three-dimensional
GLS 3D	left ventricle 3D global longitudinal strain
GCS 3D	left ventricle 3D global circumferential strain
GRS 3D	left ventricle 3D global radial strain
EFS	event-free survival
OS	overall survival
CTRCD	cancer therapy related cardiac dysfunction
CCM	chemotherapy-induced cardiomyopathy
2D-STE	two-dimensional speckle-tracing echocardiography
3D-STE	three-dimensional speckle-tracking echocardiography
LVEF 3D	left ventricle ejection fraction 3D
EDV 3D	end diastolic volume 3D
ESV 3D	end systolic volume 3D
ESVI 3D	end systolic volume indexed 3D
min	minimum
max	maximum
WBC	white blood cells
AIEOP BMF 2017	international collaborative treatment protocol for children and adolescents with acute lymphoblastic leukemia
ALL IC-BMF 2009	Randomized Trial of the I-BFM-SG for the Management of Childhood non-B Acute Lymphoblastic Leukemia
M1/2/3	marrow, <5% blasts (M1); marrow ≥5 to <25% blasts (M2), marrow, ≥25% blasts (M3)
BSA	body surface area
BMI	body mass index
